# Impact of climate change on temperature variations and extrinsic incubation period of malaria parasites in Chennai, India: implications for its disease transmission potential

**DOI:** 10.1186/s13071-024-06165-0

**Published:** 2024-03-15

**Authors:** P. K. Kripa, P. S. Thanzeen, Nagaraj Jaganathasamy, Sangamithra Ravishankaran, Anupkumar R. Anvikar, Alex Eapen

**Affiliations:** 1 Field Unit, ICMR-National Institute of Malaria Research, Chennai, India; 2 Academy of Scientific and Innovative Research (AcSIR), Ghaziabad, India; 3 ICMR-National Institute of Immunohaematology, Chandrapur Unit, Chandrapur, Maharashtra India; 4 ICMR-National Institute of Malaria Research, Sector 8, Dwarka, New Delhi India

**Keywords:** Extrinsic incubation period, Degree-day model, Daily temperature range, *Anopheles stephensi*

## Abstract

**Background:**

The global temperature has significantly risen in the past century. Studies have indicated that higher temperature intensifies malaria transmission in tropical and temperate countries. Temperature fluctuations will have a potential impact on parasite development in the vector *Anopheles* mosquito.

**Methods:**

Year-long microclimate temperatures were recorded from a malaria-endemic area, Chennai, India, from September 2021 to August 2022. HOBO data loggers were placed in different vector resting sites including indoor and outdoor roof types. Downloaded temperatures were categorised by season, and the mean temperature was compared with data from the same study area recorded from November 2012 to October 2013. The extrinsic incubation period for *Plasmodium falciparum* and *P. vivax* was calculated from longitudinal temperatures recorded during both periods. Vector surveillance was also carried out in the area during the summer season.

**Results:**

In general, temperature and daily temperature range (DTR) have increased significantly compared to the 2012–2013 data, especially the DTR of indoor asbestos structures, from 4.30 ℃ to 12.62 ℃ in 2021–2022, unlike the marginal increase observed in thatched and concrete structures. Likewise, the average DTR of outdoor asbestos structures increased from 5.02 ℃ (2012–2013) to 8.76 ℃ (2021–2022) although the increase was marginal in thatched structures and, surprisingly, showed no such changes in concrete structures. The key finding of the extrinsic incubation period (EIP) is that a decreasing trend was observed in 2021–2022 compared to 2012–2013, mainly in indoor asbestos structures from 7.01 to 6.35 days, which negatively correlated with the current observation of an increase in temperature. Vector surveillance undertaken in the summer season revealed the presence of *Anopheles* breeding in various habitats. *Anopheles stephensi* could be collected using CDC light traps along with other mosquito species.

**Conclusion:**

The microclimate temperature has increased significantly over the years, and mosquitoes are gradually adapting to this rising temperature. Temperature negatively correlates with the extrinsic incubation period of the parasite. As the temperature increases, the development of the parasite in *An. stephensi* will be faster because of a decrease in EIP, thus requiring relatively fewer days, posing a risk for disease transmission and a hindrance to malaria elimination efforts.

**Graphical Abstract:**

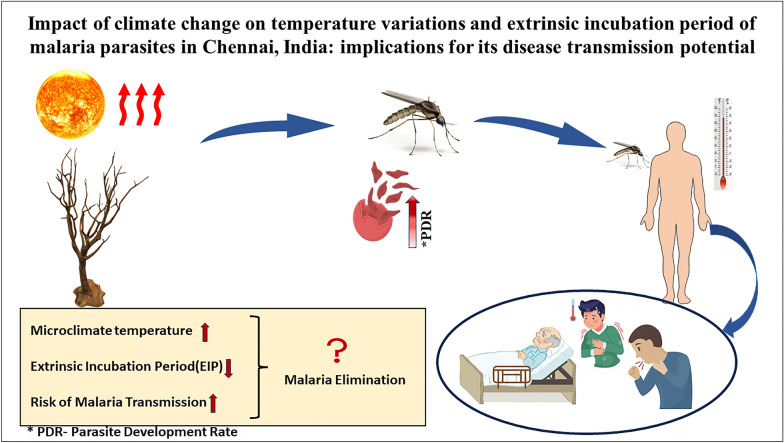

## Background

Despite the global efforts towards malaria elimination, around 63,000 deaths were reported globally between 2019 and 2021, mainly due to disruption to essential, malaria-related services during the COVID-19 pandemic, increasing the need to accelerate efforts to eliminate the disease [1]. A new challenge arising in this scenario is the spread of *Anopheles stephensi*, native to South Asia and parts of the Arabian Peninsula, to Djibouti (2012), Ethiopia and Sudan (2016), Somalia (2019), Nigeria (2020), Yemen (2021) and Ghana, and Kenya (2022) [1]. This invasion of *An. stephensi* in sub-Saharan Africa, where the burden of malaria is the highest and > 40% of the population lives in urban environments, is a matter of grave concern. *Anopheles stephensi* is notorious as an urban malaria vector, and global urbanization adds to the threat of the spread of the disease [1]. Moreover, shifts in climate conditions within these regions may alter habitats that are typically unfavourable for malaria-transmitting mosquitoes or temporarily lengthen the period during which people are vulnerable to malaria [2]. WHO has launched an initiative to halt the continued expansion of *An. stephensi* in Africa. To bolster an efficient regional reaction, WHO has put forth a comprehensive five-part strategy. This includes fostering greater collaboration, enhancing surveillance efforts, improving the exchange of information, creating guidance, and prioritising research [3].

The transmission of malaria is highly influenced by the dynamic environmental temperature and parasites and mosquitoes that are exposed to the variations in daily temperature. Earlier studies confirmed that the mean temperature is highly influenced by these variations [4]. Empirical evidence is available showing that along with mean temperatures, daily fluctuations in temperature also affect parasite infection, the rate of parasite development, and the essential elements of mosquito biology. These factors play a huge role in disease transmission intensity [5], dispersion, and distribution of the vectors and patterns of disease transmission that are known to be highly influenced by the changing climatic conditions [6, 7]. The temperature variations affect the length of the gonotrophic cycle, fecundity, biting rate, longevity, and development of immature mosquitoes [8]. Sporogonic development of the parasite in the vector is also affected by variables such as temperature, relative humidity, and rainfall [9]. Even negligible alterations in mean or diurnal temperature can result in significant variations in the life cycle of both vector and parasite, which eventually determine transmission intensity [10].

It has been hypothesised that a rise in temperature above the average range would not only aid in the selection of temperature-tolerant mosquitoes in a population but also affect both intrinsic and extrinsic factors that have direct implications for disease transmission, survival rates, and vectorial capacity [11]. Considering the global rise in temperature and the rapid spread of *An. stephensi* to African countries, the impact of temperature on the vectors is a matter of concern and needs in-depth investigations to understand its disease transmission potential. The extrinsic incubation period (EIP) of the parasite (time required for development within a mosquito and becoming infectious) is one such factor that determines the transmission potential of the disease [12]. As temperature plays a role in the EIP, the alarming increase in temperature due to global warming will have a significant impact on the EIP of malaria parasites.

The current study was conducted in the city of Chennai in Tamil Nadu, Southeast India, where the major vector of malaria is *An. stephensi*. It has been reported that this species rests in both indoor and outdoor environments [10]. The study aims to analyse the impact of changes in temperature, both indoor and outdoor resting sites of the vector, and the effect of temperature on the EIP of parasites by recording year-long temperature data using HOBO data loggers (U10-003). The study also analysed the variations in temperature and EIP over a gap of 10 years, comparing them with our previously published study data from 2012–2013 [13].

## Methods

### Study site and sampling method

Since the study was focused on the variations in temperature over 10 years, the same study area of 2012–2013 was selected to avoid bias due to site/area-based fluctuations [13]. The region covered by Besant Nagar clinic (13.0002°N, 80.2668°E) was selected previously based on the malaria prevalence during the 2006–2012 period obtained from the Regional Office for Health and Family Welfare (ROH & FW) at Besant Nagar, Chennai. Suitable human dwellings were selected after obtaining the necessary consent for year-long environmental monitoring of the micro-climatic temperature of various roof types in indoor and outdoor environments.

### Recording the microclimatic temperature and relative humidity (RH)

Year-long microclimate data of ambient, atmospheric temperature from the preferred resting sites of adult *An. stephensi* were recorded from Besant Nagar, Chennai, using temperature and relative humidity data loggers (Onset HOBO U10-003) from September 2021 to August 2022. The data loggers were launched using HOBOWARE Lite (version 1.2.3) software [13] and were placed in three different resting sites, which include various indoor and outdoor roof types: thatched, asbestos, and concrete structures. A total of 18 HOBO data loggers were placed with three replicates for each structure type (indoor as well as outdoor). Both indoors and outdoors, HOBO data loggers were attached to the wall or horizontal flat surface 1–2 feet down from the roof after obtaining consent from household members. Data loggers were carefully placed away from places such as the kitchen, ventilators, bathrooms, etc., to avoid errors/discrepancies in temperature data reading. The launching date and time of the HOBO data logger, data collection site with address, geo-coordinates, habitat type (household roof characteristics), and other relevant information were recorded. Field visits were undertaken fortnightly, and temperature and relative humidity readings were downloaded onto a laptop using the software. During these visits, the data loggers with low battery levels were replaced to ensure continuous data recording. After the readings were downloaded, they were fixed in the same place to continue recording to obtain year-long data. The geo-coordinates of the resting habitats along with altitude data were recorded using Garmin GPS (version 2.40).

### Vector surveillance

#### *Immature*

The immature surveillance was undertaken in malaria-endemic areas focusing on the anopheline breeding habitats (both intra- and peri-domestic) and natural aquatic habitats where *Anopheles* mosquitoes preferably breed in houses/apartments and their premises during the summer season. Since we were focusing on the impact of high temperatures on vectors, mainly *An. stephensi*, the surveillance was conducted during this season. Immature sampling was undertaken following standard/appropriate sampling techniques such as dipping, bucketing, and well net sampling methods [14]. The larval sampling was done twice a month. The collected samples were transferred to properly labelled plastic containers and then carefully transported to the laboratory to avoid mortality. The collected immatures were reared in the laboratory in standard conditions (27 °C and 80% RH). The mosquito species that emerged from the collected samples were identified morphologically using standard mosquito identification keys [15].

#### *Adult*

Adult surveillance was conducted using mechanical/oral aspirators and flashlights to estimate the density in the study sites by undertaking resting collections, pyrethrum spray sheet (PSC) and light trap collections from March to May 2022 (summer season). Indoor resting collections were undertaken during dawn in the appropriate houses and cattle sheds in the area. Pyrethrum spray sheet catches were done to estimate the number of mosquitoes resting indoors where people had slept the previous night during the morning hours before the households started cooking. Thatched/tiled or asbestos houses with separate bedrooms were selected, depending on the availability of such houses in the area for PSCs to collect the maximum number of indoor resting mosquitoes. Light traps were placed indoors near the host by hanging them ~ 1.8 m from the ground to collect anophelines [16].

A total of 10 resting (eight human dwellings, one cattle shed, and one outdoor), three pyrethrum spray sheet, and three light trap collections (from two households and one cattle shed) were carried out in both areas. The collected adult mosquitoes were kept in test tubes/plastic containers depending on the density and labelled with the date of collection. All the mosquito samples were brought to the laboratory in temperature-controlled conditions and the live mosquitoes were kept in thermocol/styrofoam boxes to prevent mortality during transportation. The mosquito species collected were identified following standard identification keys [15].

### Data analysis

The downloaded data points were arranged and categorised into four seasons, namely winter (December–February), summer (March–May), pre-monsoon (June- August), and monsoon (September–November), as experienced in the study area. The monthly mean temperature and DTR were calculated for all the months. Microenvironmental data were statistically analysed in IBM SPSS Statistics, version 23. All the data points were checked for normality using the Shapiro-Wilk test. Differences in temperature and DTR of different structure types for all seasons during the 2021–2022 period were statistically analysed by one-way ANOVA. Since the ANOVA results were significant, a post hoc test was performed to identify the data set that contributed to the significant results. The data obtained during 2021–2022 were then compared with the data from the same study area from 2012 to 2013 of our previously published study [13] using paired t-tests. The microenvironmental temperature was then compared with the macroenvironmental temperature obtained from https://power.larc.nasa.gov [17]. The monthly average precipitation data were obtained from https://power.larc.nasa.gov [17] to analyse the recorded relative humidity.

### Extrinsic incubation period for *Plasmodium vivax* and *P. falciparum*

The season-wise EIP for the development of *Plasmodium* in mosquitoes was calculated using Detinova’s degree-day model [18]. In the model, the sum of heat in degree-days required for completing a sporogonic cycle is 105 °C and 111 °C for *Plasmodium vivax* and *P. falciparum*, respectively. The sum of heat is the total number of degree-days in the given period. A degree-day (the degree-24 h) is the number of degrees by which the mean temperature of the day concerned exceeds the lower threshold temperature for the development of the organism of the given species, i.e. the temperature below which development does not occur [19, 20]. The EIP based on this method was calculated using the formula EIP = 111/(T-16) for *P. falciparum*, where 111 indicates the degree-days and Tmin = 16, and for *P. vivax* the EIP = 105/(T-14.5), where 105 indicates the degree-days for *P. vivax* and Tmin = 14.5 [18]. Pearson correlation analysis was performed to investigate the relationship between average temperature and EIP for *P. vivax* and *P. falciparum.*

## Results

### Diversity of seasonal temperature profiles in indoor and outdoor environments of different roof types during 2021–2022

**Fig. 1:**
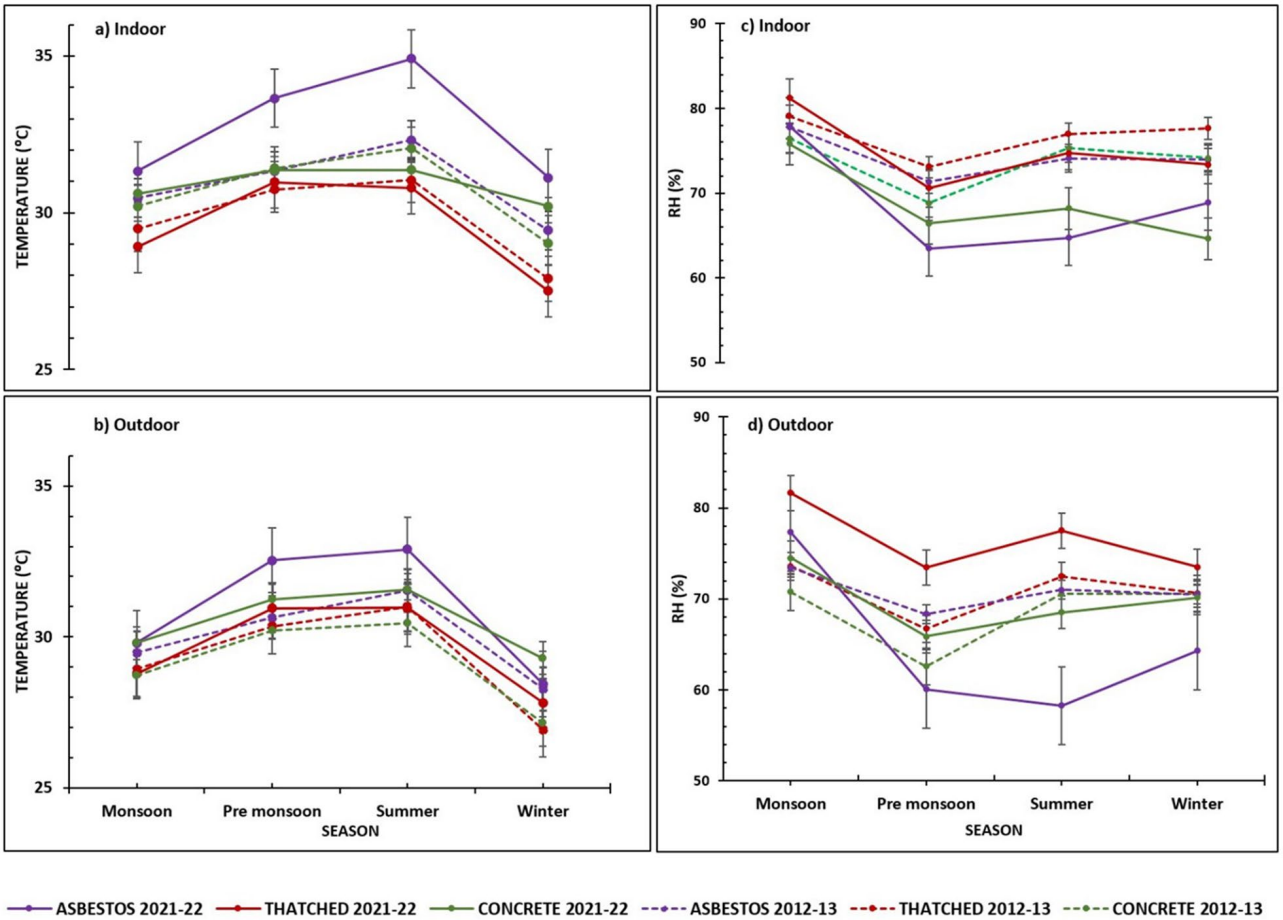
Season-wise mean temperature and relative humidity recorded from different roof types in 2012–2013 and 2021–2022. **a** Mean indoor temperature. **b** Mean outdoor temperature. **c** Mean indoor RH. **d** Mean outdoor RH

The indoor and outdoor temperatures of concrete and thatched roof structures did not show any significant difference (*p* = 0.96) during the pre-monsoon season. Similarly, the outdoor temperature for concrete and asbestos roof types did not vary (*p* = 1.00) during the monsoon season. All other roof types showed significant differences in temperature for all other seasons (Fig. 1).

For the indoor temperature of the roof types, the highest temperature was observed in asbestos (34.92 ± 1.78) in summer and the lowest in thatched (27.90 ± 0.97) during winter (Fig. 1). For the outdoor temperature of all roof types, asbestos had the highest temperature in all seasons except winter. However, during winter, outdoor temperatures for concrete roof types were higher (29.30 ± 1.08). However, the outdoor temperature for thatched roof types remained lower throughout all the seasons.

### Seasonal variations in temperature and relative humidity across various roof types between 2012–2013 and 2021–2022

Comparing the 2021–2022 temperature data of the winter season with those of 2012–2013, there was a significant increase in the temperature during 2021–2022, for both indoor and outdoor temperatures of all roof types, except the indoor temperature of thatched structures (Fig. 1) (Table 1). For thatched roofs, indoors showed a significant decrease in temperature (*p* = 0.00) during 2021–2022 (27.50 °C ± 0.97) compared to 2012–2013 (27.90 °C ± 0.51).

**Table 1:** Mean temperature and daily temperature range in various structure types in all four seasons

	Structure	Indoor/outdoor	Temperature (°C)	Winter	Summer	Pre-monsoon	Monsoon
2021 Sep–2022 Aug	Asbestos	Indoor	Mean DTR	31.11 ± 1.62 13.37	34.92 ± 1.79 16.37	33.65 ± 1.75 11.47	31.33 ± 2.93 9.26
		Outdoor	Mean DTR	28.44 ± 1.17 7.82	32.91 ± 1.90 10.46	32.53 ± 1.45 9.69	29.79 ± 2.45 7.06
	Thatched	Indoor	Mean DTR	27.50 + 0.96 5.36	30.79 ± 1.10 5.35	30.97 ± 0.97 6.57	28.92 ± 1.78 4.92
		Outdoor	Mean DTR	27.81 ± 0.82 5.03	30.97 ± 1.24 5.62	30.95 ± 0.96 6.87	28.78±1.71 5.04
	Concrete	Indoor	Mean DTR	30.20 ± 0.65 0.97	31.37 ± 0.46 3.56	31.35 ± 0.79 4.35	30.61 ± 0.94 2.90
		Outdoor	Mean DTR	29.30 ± 1.08 5.02	31.56 ± 1.16 3.62	31.24 ± 0.97 5.18	29.79 ± 2.10 4.03
2012 Nov–2013 Oct	Asbestos	Indoor	Mean DTR	29.44 ± 0.56 4.15	31.99 ± 0.98 4.21	31.33 ± 1.72 4.83	30.47 ± 1.36 3.99
		Outdoor	Mean DTR	28.27 ± 0.84 4.86	31.02 ± 1.05 4.97	30.64 ± 1.70 5.28	29.47 ± 1.53 4.95
	Thatched	Indoor	Mean DTR	27.90 ± 0.51 3.95	31.28 ± 1.23 4.31	30.74 ± 1.63 4.03	29.48 ± 1.39 4.02
		Outdoor	Mean DTR	26.92 ± 0.47 5.13	30.66 ± 1.51 6.56	30.35±1.80 5.44	28.93±1.48 4.34
	Concrete	Indoor	Mean DTR	29.01 ± 0.43 1.84	32.61 ± 1.01 2.04	31.42 ± 1.46 1.92	30.21 ± 1.13 1.90
		Outdoor	Mean DTR	27.14 + 0.48 4.62	30.03 ± 1.36 5.50	30.21 ± 1.56 4.29	28.72 ± 1.25 3.43

*DTR* daily temperature range

In the summer season, all roof types were warmer in 2021–2022 except for the indoor temperature of the concrete roofs (31.36 °C  ± 0.47). Concrete structures were warmer in 2012–2013 (32.06 °C ± 1.01). The outdoor temperature of the thatched structures did not show any significant difference in temperature compared to the temperature profile between 2012–2013 and 2021–2022 (Fig. 1) (Table 1). Similarly, in the pre-monsoon season, all the roof types were warmer during 2021–2022 except the indoor temperature of concrete and thatched structures. There was no significant difference between the means in 2012–2013 and 2021–2022 for the indoor concrete (*p* = 0.68) and thatched structures (*p* = 0.23).

In the monsoon season, the outdoor temperatures for the thatched roof type did not show any significant change in temperature in 2012–2013 and 2021–2022. Nevertheless, the other two roof types were warmer in 2021–2022 during the monsoon season. Comparing the indoor temperatures in the monsoon season, all the structures were warmer in 2021–2022 except the thatched roof type. The indoors of the thatched roof structures were warmer in 2012–2013.

In general, there was a significant decrease in relative humidity during 2021–2022 compared to 2012–2013 for indoors of all roof types across all seasons except the indoors of thatched structure wherein the RH increased in the monsoon season. For outdoors, the RH increased in thatched roof type across all seasons in 2021–2022. Furthermore, the RH recorded in the outdoors of concrete structures increased for monsoon and pre-monsoon seasons during 2021–2022. Similarly, the outdoors of asbestos roofs also showed increased RH during the monsoon season in 2021–2022. (Fig. 1).

The minimum humidity for the asbestos roof type (60.09 ± 15%) was recorded indoors during 2021–2022 in the pre-monsoon season, unlike in 2012–2013, when it was observed indoors for the concrete roof type (68.82 ± 8.48%) during the same season. Nonetheless, the relative humidity observed outdoors was different. The minimum humidity was recorded in the summer season for the outdoor asbestos (64.70 ± 12.46%) roof type during 2021–2022, while in 2012–2013 it was observed outdoors for the concrete (68.81 ± 8.48%) roof type in the pre-monsoon season (Fig. 1).

### Vector surveillance (2021–2022)

Mosquito breeding was observed in plastic overhead tanks (pOHT), cemented overhead tanks (cOHT), wells, cement tanks, curing pits, barrels, discarded mud pots, discarded aluminium vessels, etc. Of 518 breeding habitats surveyed, 79 (15.2%) were positive for anophelines. Among the surveyed habitats, the highest water temperature recorded was 36.2 °C, which was observed in stagnant water on a terrace. The adults that emerged from the immature collections were *An. stephensi*. In adult collections, only one *An. stephensi* was collected in the resting collection along with other species such as *Culex quinquefasciatus, Stegomyia aegypti, Cx. gelidus*, and *Cx. tritaeniorhynchus*. Surprisingly, no anophelines were collected in the pyrethrum spray sheet collections. However, in light trap collections, *An. stephensi* (8), *An. pallidus* (3), and *An. subpictus* (1) were collected besides, *Cx. tritaeniorhynchus* and *St. aegypti*.

### Difference between macro- and microenvironmental temperature profiles

The average daily temperatures recorded from data loggers within the local transmission sites were significantly warmer than the data obtained from https://power.larc.nasa.gov for both periods, 2012–2013 and 2021–2022, indicating the importance of microclimatic variables in vector resting habitats.

### Comparison of the daily temperature range in 2012–2013 and 2021–2022

**Fig. 2:**
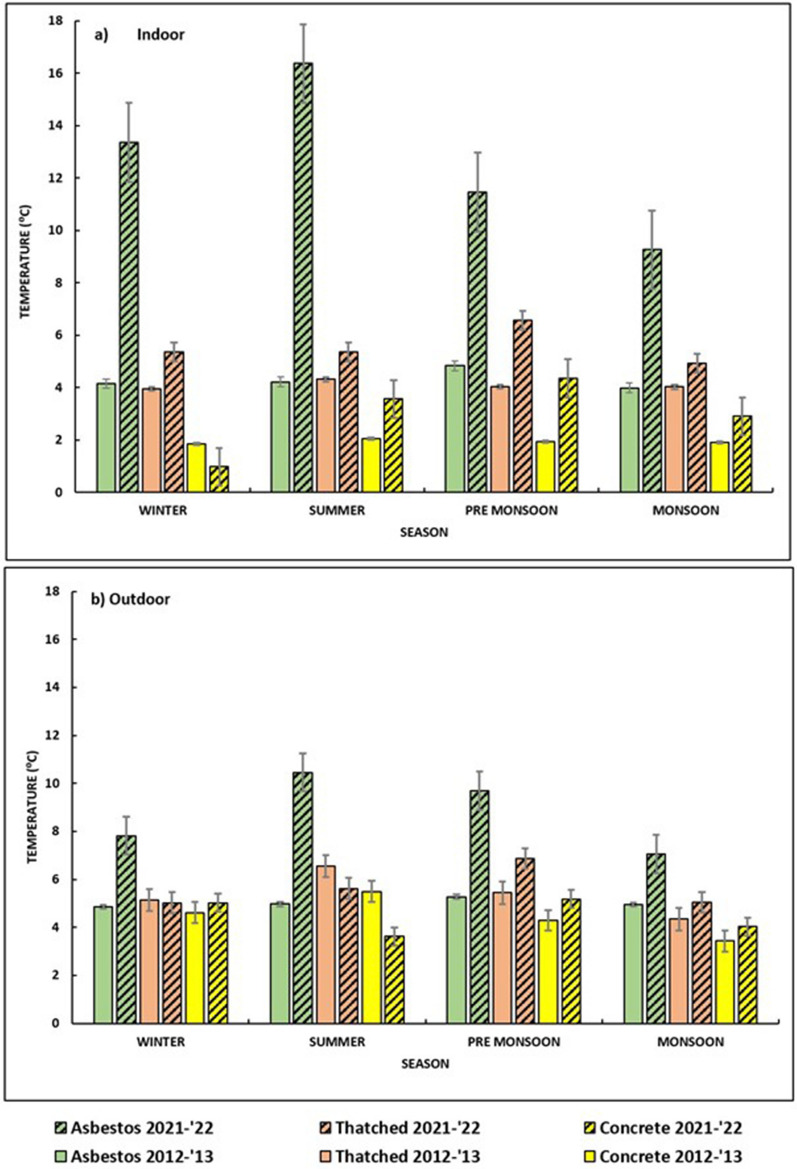
Daily temperature range (DTR) observed in various roof types in 2012–2013 and 2021–2022. **a** Indoor, **b** outdoor

The daily temperature range showed a significant increase during 2021–2022 compared to 2012–2013 data for all the structure types in all seasons except for the indoor concrete structure in the winter season. The average DTR for indoor asbestos structures increased drastically from 4.30 °C in 2012–2013 to 12.62 °C in 2021–2022. However, for the indoor thatched structures, the increase in DTR was marginal from 4.08 °C in 2012–2013 to 5.55 °C in 2021–2022. Similarly, for indoor concrete structures, the average DTR increased from 1.93 °C in 2012–2013 to 2.95 °C in 2021–2022. The DTR was lower in 2021–2022 in the winter season for concrete structures (Fig. 2). The DTR showed a steady pattern for the indoors of all the roof types in 2012–2013, but it fluctuated throughout the seasons when in the 2021–2022 period. Furthermore, a broader spectrum of DTR could be noted within the interior of asbestos buildings, and this variation was conspicuous in 2021–2022 (Fig. 2).

The DTR of all roof types for outdoors showed an increase in general for 2021–2022 except for concrete structures. The average DTR for outdoor asbestos structures increased from 5.02 °C in 2012–2013 to 8.76 °C in 2021–2022. However, in the outdoors of thatched structures, the DTR showed a marginal increase from 5.37 °C in 2012–2013 to 5.64 °C in 2021–2022. Nevertheless, for the outdoor concrete structures, the average DTR did not show any change over the years (4.46 °C).

### Variations in the extrinsic incubation period of *Plasmodium vivax* and *P. falciparum* across various roof types

**Fig. 3:**
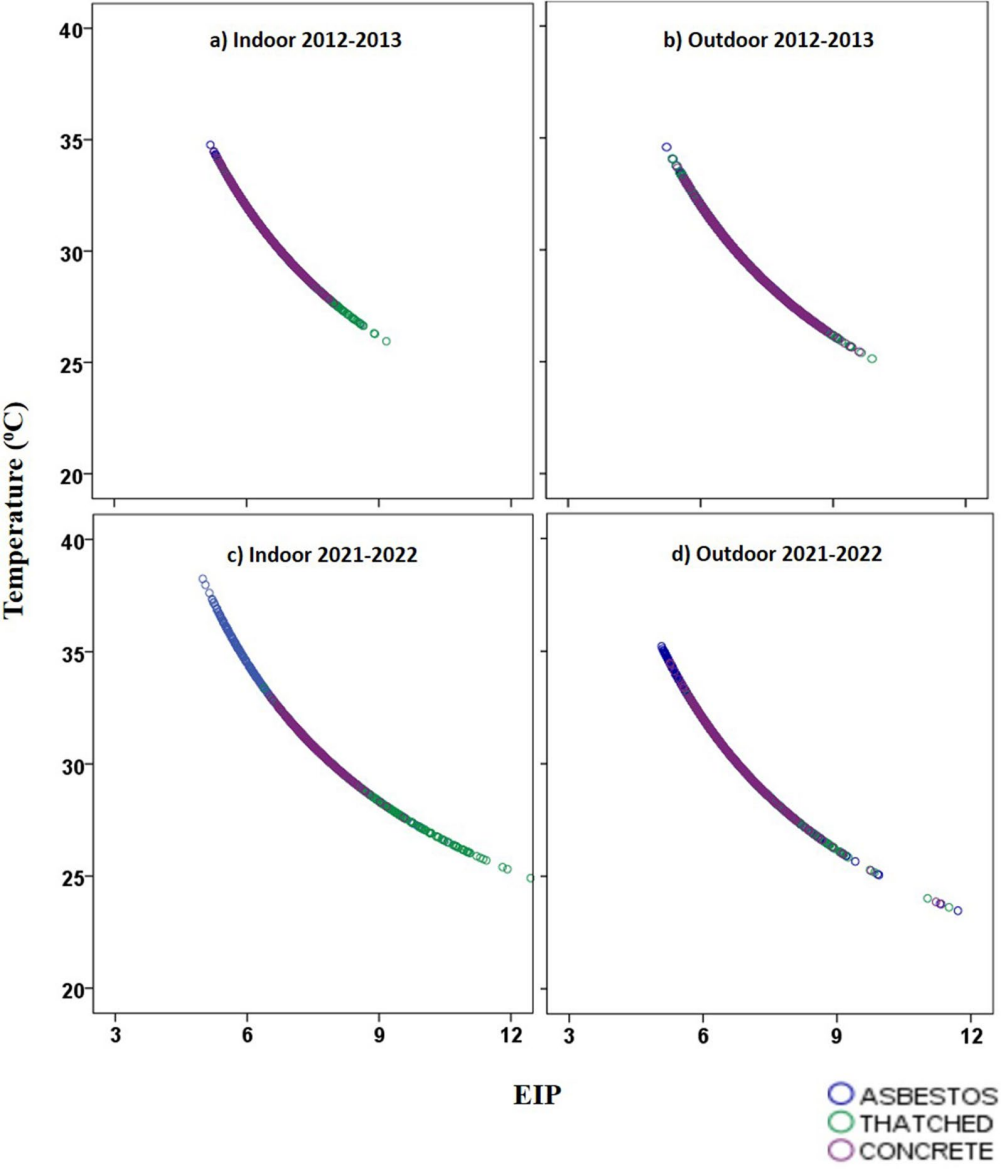
Correlation between mean temperature and extrinsic incubation of *Plasmodium* in **a** indoor structure type 2012–2013, **b** outdoor structure type 2012–2013, **c** indoor structure type 2021–2012, **d** outdoor structure type 2021–2022

In general, when we compared the EIP for *P. vivax* and *P. falciparum* for 2012–2013 and 2021–2022, the EIP showed a decreasing trend, which negatively correlated with our observation of an increase in temperature from 2012–2013 to 2021–2022 (Fig. 3) (Table 2). The average EIP for indoor asbestos structures decreased from 7.01 days in 2012–2013 to 6.35 days in 2021–2022. However, for indoor thatched structures, EIP showed a slight increase from 7.57 days in 2012–2013 to 7.74 days in 2021–2022. Likewise, the average EIP decreased from 7.10 days in 2012–2013 to 6.96 in 2021–2022 inside concrete structures

**Table 2:** Seasonal extrinsic incubation period of *Plasmodium vivax* and *P. falciparum* in different indoor and outdoor structure types (mean, minimum, and maximum)

Year	Structure	Indoors/outdoors	Winter mean (min–max)		Summer mean (min–max)		Pre-monsoon mean (min–max)		Monsoon mean (min–max)	
			*Pv*	*Pf*	*Pv*	*Pf*	*Pv*	*Pf*	*Pv*	*Pf*
2021 Sep–2022 Aug	Asbestos	Indoor	6.38 (5.27–8.17)	7.43 (6.03–9.78)	5.19 (4.42–7.25)	5.93 (4.99–8.56)	5.53 (4.47–6.71)	6.35 (5.05–7.85)	6.45 (4.85–10.70)	7.54 (5.51–13.35)
		Outdoor	7.59 (6.39–9.91)	9.01 (7.43–12.21)	5.78 (5.09–7.64)	6.66 (5.81–9.06)	5.86 (5.07–6.97)	6.77 (5.77–8.18)	7.07 (5.43–11.71)	8.34 (6.23–14.86)
	Thatched	Indoor	8.12 (7.21–10.30)	9.72 (8.49–12.77)	6.48 (5.56–7.74)	7.55 (6.38–9.19)	6.40 (5.73–7.40)	7.45 (6.59–8.75)	7.41 (6.04–11.54)	8.78 (6.99–14.61)
		Outdoor	7.92 (7.21–9.84)	9.45 (8.50–12.11)	6.41 (5.56–7.66)	7.47 (6.38–9.10)	6.41 (5.72–7.31)	7.46 (6.58–8.62)	7.47 (6.12–11.51)	8.87 (7.09–14.56)
	Concrete	Indoor	6.70 (6.23–7.35)	7.83 (7.22–8.68)	6.23 (5.87–6.73)	7.23 (6.78–7.87)	6.24 (5.66–6.89)	7.25 (6.51–8.08)	6.54 (5.82–8.01)	7.63 (6.71–9.56)
		Outdoor	7.13 (6.21–8.57)	8.40 (7.20–10.33)	6.19 (5.26–7.41)	7.18 (6.01–8.76)	6.29 (5.72–7.25)	7.31 (6.58–8.55)	7.02 (5.57–11.34)	8.27 (6.39–14.31)
2012 Nov–2013 Oct	Asbestos	Indoor	7.04 (6.66–7.96)	8.27 (7.78–9.50)	5.91 (5.18–6.85)	6.83 (5.91–8.03)	6.31 (5.26–8.58)	7.33 (6.01–10.33)	6.63 (5.79–8.44)	7.75 (6.67–10.15)
		Outdoor	7.65 (6.83–9.10)	9.09 (8.00–11.05)	6.19 (5.23–7.80)	7.18 (5.97–9.28)	6.58 (5.53–9.08)	7.69 (6.35–11.02)	7.09 (5.94–9.39)	8.35 (6.87–11.47)
	Thatched	Indoor	7.85 (7.40–8.91)	9.35 (8.75–10.79)	6.38 (5.35–7.62)	7.43 (6.13–9.04)	6.53 (5.48–8.89)	7.62 (6.28–10.76)	7.07 (6.13–9.17)	8.32 (7.10–11.15)
		Outdoor	8.47 (7.82–9.63)	10.19 (9.31–11.81)	6.42 (5.36–8.25)	7.48 (6.14–9.89)	6.72 (5.53–9.40)	7.87 (6.35–11.47)	7.36 (6.24–9.88)	8.70 (7.25–12.16)
	Concrete	Indoor	7.24 (6.78–7.87)	8.54 (7.94–9.37)	6.00 (5.26–6.90)	6.94 (6.01–8.09)	6.25 (5.35–7.98)	7.26 (6.13–9.52)	6.72 (5.93–7.85)	7.86 (6.85–9.34)
		Outdoor	8.32 (7.70–9.59)	9.99 (9.14–11.75)	6.63 (5.48–8.16)	7.75 (6.28–9.76)	6.75 (5.61–8.88)	7.91 (6.45–10.75)	7.44 (6.44–9.27)	8.82 (7.50–11.30)

During the pre-monsoon season, both indoor and outdoor EIPs of all the structure types showed a decrease in 2021–2022, indicating that parasite development now occurred much faster than in 2012–2013. In winter, summer, and monsoon seasons, the indoor EIP for thatched structures showed an increase which correlated with the decrease in temperature during this period. In monsoon season, the outdoor EIP increased for thatched structures, indicating that in thatched structures the parasite development rate was generally slower owing to the decrease in temperature. During summer, the indoor EIP for concrete structures showed an increase in 2021–2022, which was again negatively correlated with the decreased indoor temperature of concrete roof types. An increase in the DTR in 2021–2022 was observed compared to the 2012–2013 data, and this observation correlated with the increased range for EIP. However, in concrete structures, except for winter, the DTR decreased, and so the EIP range for this structure was narrow.

## Discussion

The current study focuses on data recording and analysis of microclimatic variables and temperature for 2021–2022 across three different structural roof types, namely, asbestos, thatched, and concrete, where the mosquitoes preferred to rest indoors. The data obtained were then compared to the microclimate data recorded in 2012–2013 from our previously published study [13] to determine the variation between microenvironment temperature variables over these years. Multiple studies have already shown that microclimatic variables are significant factors in disease transmission as opposed to overall weather station data recorded far from the study site [10, 13]. This study showed that the local transmission site is warmer by at least 3–4 °C compared to the weather station data recorded and analysed. A similar observation was noted in our previously published study [13].

Generally, it has been observed that, over the years, there has been a significant increase in both indoor and outdoor temperatures across all roof types indicating the impact of global warming. Studies have already reported that, in tropical countries, an increase in temperature carries an increased risk of malaria burden due to global warming [10]. Since Chennai features a tropical dry and wet climate, the current study with a warmer environment is a matter of concern when it comes to disease transmission potential. With the increased temperature, a question may arise whether the vector will survive in higher temperatures or not. In vector surveillance undertaken in the same area, there were a few habitats with immature anophelines during summer indicating that vectors do survive in higher temperatures. However, in adult collections, *An. stephensi* was rarely collected, but other mosquito species such as *Cx. quinquefasciatus, Cx. gelidus*, and *St. aegypti* were collected. A few studies reported that, over the years, the density of adult *An. stephensi* has declined drastically with fewer breeding habitats and relatively low breeding in habitats/sources due to habitat manipulation and vector interventions unlike in earlier years. Furthermore, ethological studies on mosquitoes have shown that in such cases mosquitoes prefer cooler areas and avoid hotter temperatures [21, 22].

Previous studies indicated that mosquito abundance and relative humidity have a weak negative correlation, that is, when RH decreases there are chances that the abundance of mosquitoes may increase (21). In the monsoon season, the average precipitation (mm/day) was observed to be higher during 2021–2022 (11.82 mm/day) than in 2012–2013 (3.51 mm/day), which was positively correlated with recorded relative humidity, that is, all roof types showed higher RH in 2021–2022 during the season. Thatched outdoor roof type showed a significant increase in RH during 2021–2022 throughout the seasons. In 2021–2022, our study sites experienced precipitation almost every month. Therefore, thatched roofs were always moist, hence the increased humidity.

DTR was relatively narrow and more stable previously, but currently, it is showing a wider range and is fluctuating more (Fig. 2). An increase in temperature and DTR has a significant impact on parasite prevalence, parasite intensity, and mortality of mosquitoes, and this has decreased overall vectorial capacity for both mosquito species, *An. stephensi* and *An. gambiae* [23].

Regarding EIP of parasites calculated based on Detinova’s degree-day model, the main observation was that when there is an increase in temperature, the EIP decreases steadily for both *P. falciparum* and *P. vivax*. In the current study, the area is experiencing a warmer climate pattern; hence, the EIP is decreasing, indicating a strong negative correlation in the study (Fig. 3). A mechanistic mathematical model aligns with our observations. According to the model, it is predicted that an increase in temperature from 21˚C to 34 °C decreased the EIP_50_ from 16.1 to 8.8 days [22]. According to the thermodynamic model, parasite development rate (PDR) is directly proportional to temperature and EIP is reciprocal to PDR [13]. Hence, when temperature increases, PDR increases, while EIP tends to decrease, which is similar to the case observed from our findings with Detinova’s degree-day model [18].

The study reiterates the importance of the ambient environmental temperature to which the vector is exposed in the resting site and the factors that influence parasite development. Previous studies have also shown that temperature affects the sporogonic development of *P. falciparum* in anophelines and the ookinete maturation rate. At lower temperatures (21–27 °C), infection rates of both ookinetes and oocysts were unaffected, but at higher temperatures (30 and 32 °C), there was a significant impact on parasite densities and infection rates because this changes the developmental processes between fertilization and ookinete production [24]. Another notable observation was a wide range of DTR with fluctuation in 2021–2022, unlike the 2012–2013 data (Table 1). For the current dataset, EIP shows a similar pattern with a wide range of days (Table 2). There is ample evidence from previous studies that even a small change in EIP for minimal days can have a drastic impact on the transmission risk. Therefore, with increased temperature and the resultant decrease in EIP, it is obvious that transmission of the disease might quickly get faster.

## Conclusion

Global warming has increased the atmospheric temperature; as a result, the same has been observed in the DTR. The EIP of parasites has a strong negative correlation with the temperature. Currently, the EIP of *P. falciparum* and *P. vivax* is decreasing. Consequently, the development of the parasite will be faster and require relatively fewer days. Current models predicting the relationship between temperature and PDR of a parasite have an upper thermal limit for temperature. However, with increasing temperature, this upper limit has to be reconsidered. In general, the impact of global warming and increasing temperatures will thereby pose a risk for disease transmission and also may foil the efforts made to eliminate malaria.

## Limitations of the study

Analysing the data showed that the DTR increased in 2021–2022 compared to 2012–2013, as did the EIP. EIP had a wide range in 2021–2022, unlike in 2012–2013. However, the implications of these observations are difficult to derive given the lack of a real-time experiment exposing infected mosquitoes to varying daily temperatures, which was not conducted in the present study.

The prevailing models explaining the role of temperature in parasite development, such as Paaijman’s model, could not be followed in this study as our higher temperature exceeds the critical higher temperature of the model. Hence, we used the degree-day model of Detinova for this study.

The data comparison showed that in 2021–2022 the EIP of the parasite decreased significantly, indicating an increased transmission potential given that parasites will take less time to develop compared to the 2012–2013 period due to an increase in temperature as temperature and EIP are negatively correlated. Due to the unforeseen COVID-19 pandemic-related circumstances, few malaria cases were reported during the study period in the same area, mainly because of the disruption of technical services.

## Data Availability

The dataset generated during and/or analysed during the current study is available from the corresponding author upon reasonable request.

## Abbreviations

DTR: Daily temperature range
EIP: Extrinsic incubation period
GPS: Global positioning system
PSC: Pyrethrum spray sheet collection
PDR: Parasite development rate
ROH & FW: Regional Office for Health and Family Welfare
